# The structure of the cyanobactin domain of unknown function from PatG in the patellamide gene cluster

**DOI:** 10.1107/S2053230X1402425X

**Published:** 2014-11-14

**Authors:** Greg Mann, Jesko Koehnke, Andrew F. Bent, Rachael Graham, Wael Houssen, Marcel Jaspars, Uli Schwarz-Linek, James H. Naismith

**Affiliations:** a Biomedical Sciences Research Complex, University of St Andrews, North Haugh, St Andrews, Fife KY16 8ST, Scotland; b Marine Biodiscovery Centre, Department of Chemistry, University of Aberdeen, Meston Walk, Aberdeen AB24 3UE, Scotland; c Institute of Medical Sciences, University of Aberdeen, Aberdeen AB25 2ZD, Scotland; d Pharmacognosy Department, Faculty of Pharmacy, Mansoura University, Mansoura 35116, Egypt

**Keywords:** cyanobactins, patellamides, PatG, RiPPs

## Abstract

Patellamides are members of the cyanobactin family of ribosomally synthesized and post-translationally modified cyclic peptide natural products, many of which, including some patellamides, are biologically active. A detailed mechanistic understanding of the biosynthetic pathway would enable the construction of a biotechnological ‘toolkit’ to make novel analogues of patellamides that are not found in nature. All but two of the protein domains involved in patellamide biosynthesis have been characterized. The two domains of unknown function (DUFs) are homologous to each other and are found at the C-termini of the multi-domain proteins PatA and PatG. The domain sequence is found in all cyanobactin-biosynthetic pathways characterized to date, implying a functional role in cyanobactin biosynthesis. Here, the crystal structure of the PatG DUF domain is reported and its binding interactions with plausible substrates are investigated.

## Abstract 2

The highly conserved domain of unknown function in the cyanobactin superfamily has a novel fold. The protein does not appear to bind the most plausible substrates, leaving questions as to its role.

## 1. Introduction   

Prokaryotic secondary metabolism creates an array of bioactive molecules, some of which can be exploited for pharmaceutical development. Cyanobactins, secondary metabolites of cyanobacteria, are a superfamily of ribosomally synthesized and post-translationally modified peptides (RiPPs) that are highly variable with a wide range of bioactivities (Sivonen *et al.*, 2010 ▶). Patellamides, eight-residue cyclic peptides that contain oxazoline, thiazoline and thiazole heterocycles and d-amino acids (Schmidt *et al.*, 2005 ▶), belong to this superfamily and have attracted considerable interest owing to the ability of patellamide D to reverse the effects of multidrug resistance in human leukaemia cells (Williams & Jacobs, 1993 ▶; Houssen & Jaspars, 2010 ▶). These natural products are too structurally complex for cost-effective *de novo* chemical synthesis, and this prevents the creation of novel and diverse analogues with desired properties. Manipulation of the natural product’s biosynthetic pathway can offer an alternative, more cost-effective, route to such analogues, but does require a comprehensive understanding of the individual enzymes involved. Moreover, the use of the isolated enzymes can allow the introduction of non-natural functionalities.

**Figure 1:**
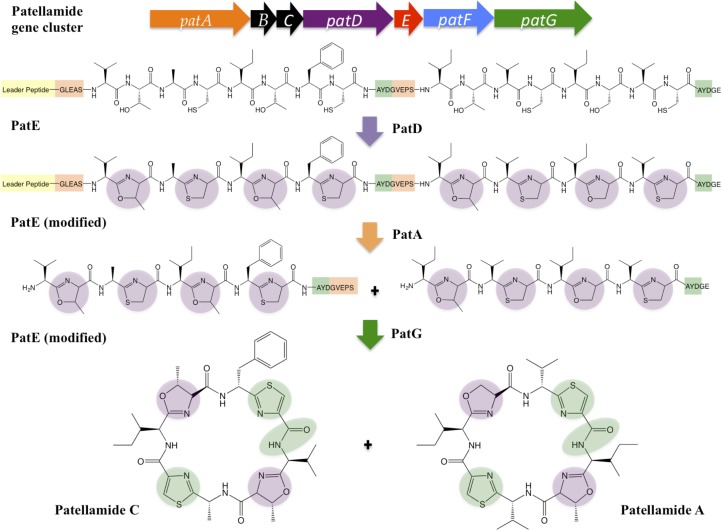
Cartoon schematic summarizing patellamide biosynthesis. All of the required biosynthetic machinery is clustered into a single operon. The precursor peptide (PatE) is processed by PatD, generating thiazoline and oxazoline heterocycles, cleaved at the N-terminus by PatA and cleaved at the C-terminus, macrocyclized and oxidized by PatG to form highly modified patellamides.

Patellamides originate from a longer precursor peptide, which is modified by a series of post-translational modifying (PTM) enzymes to yield the final cyclic product. Their biosynthesis can be attributed to a single operon (*patA*–*patG*) encoding the precursor peptide (PatE) and the enzymes required for processing (PatA, PatD and PatG; Schmidt *et al.*, 2005 ▶; Fig. 1 ▶). The precursor peptide includes a core peptide (or multiple core peptides) that becomes the natural product, as well as conserved recognition sequences and an N-terminal leader peptide, which are discarded during maturation but are required to direct post-translational tailoring. It is assumed, but has not been proven, that all of the chemical tailoring steps are required for biological activity. All but one of the chemical transformations for the tailoring steps have been assigned to domains within the processing enzymes (Agarwal *et al.*, 2012 ▶; Schmidt *et al.*, 2005 ▶). The first step is the heterocyclization of PatE core peptide cysteines, serines and threonines by PatD. This is followed by removal of the N-terminal recognition elements and leader peptide by the protease domain of PatA. Finally, PatG catalyzes the macrocyclization of the modified core peptide, simultaneously removing the C-terminal recognition sequence. Additionally, PatG is responsible for the oxidation of thiazolines to thiazoles (Fig. 1 ▶). Structural and mechanistic insights have been reported for the protease domain of PatA (Houssen *et al.*, 2012 ▶; Agarwal *et al.*, 2012 ▶), the macrocyclization domain of PatG (Koehnke *et al.*, 2012 ▶; Agarwal *et al.*, 2012 ▶), the heterocyclase TruD (Koehnke *et al.*, 2013 ▶; a close homologue of PatD from the trunkamide pathway) and the apparently inactive prenyl transferase PatF (Bent *et al.*, 2013 ▶). Only epimerization (which is likely to precede thiazoline oxidation) is unassigned to a protein domain, but it has been suggested that this could be chemically spontaneous (Milne *et al.*, 2006 ▶). Whether the epimerization and oxidation occur in the linear or the macrocyclic form is unknown. Two gene products, PatB and PatC, have been reported as non-essential to synthesis, and their role is unknown (Schmidt *et al.*, 2005 ▶).

**Figure 2:**
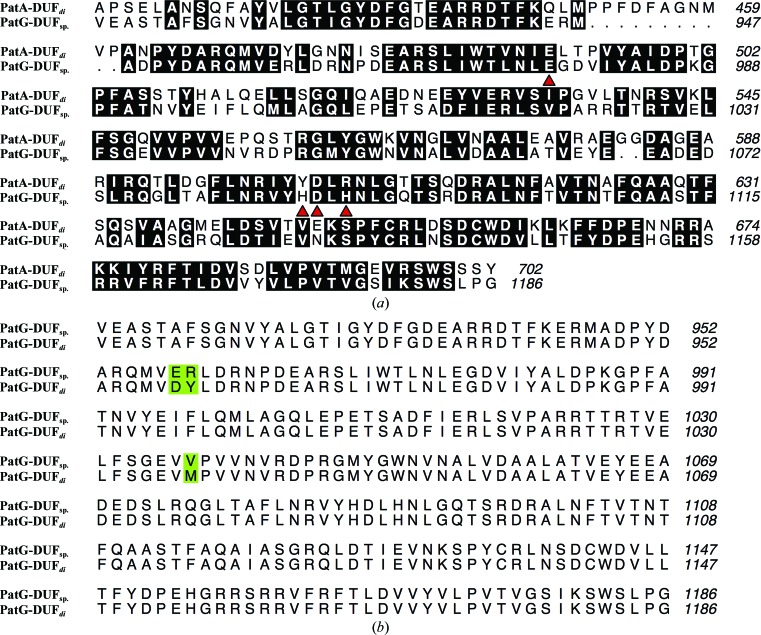
(*a*) Sequence alignment of PatA-DUF and PatG-DUF_sp._. The conserved C-terminal DUFs of PatA and PatG share 56% identity. Residues found to coordinate Zn^2+^ in PatG-DUF_sp._ are identified by red triangles. (*b*) Sequence alignment of PatG-DUF cloned from DNA isolated from *Prochloron* sp. (PatG-DUF_sp._) and PatG-DUF isolated from *P. didemni* (PatG-DUF*_di_*). Sequence differences are highlighted in green.

PatA and PatG both contain C-terminal domains of unknown function (DUFs) and these domains are homologous (56% identical) to each other (Fig. 2 ▶ *a*). The gene encoding PatG-DUF (amino-acid residues 914–1191) was amplified from gDNA isolated from *Prochloron* sp. that were isolated from Pacific reef samples. The exact strain could not be determined and the amino-acid sequence of PatG-DUF (PatG-DUF_sp._) differs at three points (E958D, R959Y and V1037M) from the deposited amino-acid sequence of PatG-DUF isolated from *P. didemni* (PatG-DUF*_di_*; accession No. AAY21556.1; Fig. 2 ▶ *b*). It is unclear as to whether these differences arose as a result of natural variance amongst species or owing to a cloning artefact.

The DUF domain is conserved in all patellamide-like cyanobactin biosynthetic pathways characterized to date (Supplementary Fig. S1^1^), suggesting it may play a functional role. To gain some insight into the DUF domain, we have determined the crystal structure of PatG-DUF_sp._ to a resolution of 1.72 Å, and using isothermal titration calorimetry (ITC) and nuclear magnetic resonance spectroscopy (NMR) we have probed its binding to some plausible substrates: the precursor peptide and intermediates formed during biosynthesis (Fig. 1 ▶).

## 2. Methods   

### 2.1. Expression of native and SeMet PatG-DUF   

The C-terminal domain of PatG from *Prochloron* sp. (PatG-DUF_sp._; amino-acid residues 914–1191) was cloned from full-length *patG* into the pHISTEV plasmid with an N-terminal His_6_ tag and a *Tobacco etch virus* (TEV) protease site (Liu & Naismith, 2009 ▶). The protein was expressed in *Escherichia coli* BL21 (DE3) cells grown in Luria–Bertani (LB) medium supplemented with 50 µg ml^−1^ kanamycin. Cultures were grown at 37°C and 200 rev min^−1^ until the OD_600_ reached 0.6; the cells were then induced by the addition of isopropyl β-d-1-thiogalactopyranoside (IPTG; final concentration 1 m*M*) and further grown at 18°C and 200 rev min^−1^ overnight.

l-Selenomethionine-labelled (SeMet) PatG-DUF_sp._ was expressed from *E. coli* BL21 (DE3) cells grown in minimal medium supplemented with glucose-free nutrient mix, 50 µg ml^−1^ kanamycin and 5% glycerol. This medium was inoculated with an overnight culture grown in LB, which was washed three times in minimal medium. After 15 min growth at 37°C, 60 mg l^−1^ l-selenomethionine was added. The cultures were returned to 37°C and 200 rev min^−1^ and grown until the OD_600_ reached 0.6, when 100 mg l^−1^ lysine, phenyl­alanine, threonine and 50 mg l^−1^ isoleucine and valine were added. After incubation for a further 20 min, expression was induced with IPTG (final concentration 1 m*M*) and the cells were grown at 18°C for 24 h. For both native and SeMet variants, the cells were harvested by centrifugation (4000*g*, 4°C, 15 min).

### 2.2. Purification of native and SeMet PatG-DUF   

Cell pellets were resuspended in lysis buffer [20 m*M* bis-tris pH 6.8, 150 m*M* NaCl, 20 m*M* imidazole, 3 m*M* β-mercaptoethanol (BME), 0.1%(*v*/*v*) Triton X-100] supplemented with 0.4 mg DNAse per gram of wet cell pellets and cOmplete EDTA-free protease-inhibitor tablets (Roche; one tablet per 50 ml resuspension). The cells were lysed *via* passage through a cell disruptor at 207 MPa (Constant Systems) and the cell debris was removed by centrifugation (40 000*g*, 4°C, 20 min). The supernatant was loaded onto a pre-equilibrated Ni–NTA column at 4°C and the column was washed with 80 column volumes of lysis buffer. The protein was eluted from the column in elution buffer [20 m*M* bis-tris pH 6.8, 150 m*M* NaCl, 250 m*M* imidazole, 3 m*M* BME, 0.1%(*v*/*v*) Triton X-100] and fractions containing protein were pooled and passed over a desalting column (Desalt 16/10, GE Healthcare) to exchange them into ‘low imidazole’ buffer (20 m*M* Tris–HCl pH 8.0, 150 m*M* NaCl, 20 m*M* imidazole, 3 m*M* BME, 10% glycerol). TEV protease was added at a mass:mass ratio of 1 mg of TEV to 10 mg of protein and the sample was incubated for 2 h at 20°C to remove the N-terminal His_6_ tag. The digested sample was passed over a second Ni–NTA column and the flowthrough was collected and loaded onto a Superdex 75 gel-filtration column (GE Healthcare) equilibrated in buffer (10 m*M* HEPES pH 7.4, 150 m*M* NaCl, 1 m*M* TCEP, 10% glycerol). The purity of the protein was determined by SDS–PAGE and its integrity was determined by mass spectrometry. Analysis of the mass spectrum of SeMet PatG-DUF_sp._ confirmed the successful incorporation of the four expected SeMet residues. Native and SeMet PatG-DUF_sp._ were concentrated to 4.75 mg ml^−1^ for crystallography.

### 2.3. Mutagenesis   

Mutagenesis of PatG-DUF_sp._ was performed using established protocols (Liu & Naismith, 2008 ▶).

### 2.4. CD spectroscopy   

Near-UV CD spectra were recorded on a Jasco J-810 spectropolarimeter with samples in 10 m*M* HEPES pH 7.4, 150 m*M* NaCl, 1 m*M* TCEP, 10% glycerol.

### 2.5. Crystallography   

Crystal screens were set up with the sitting-drop vapour-diffusion method using a Gryphon robot (Art Robbins). The protein was screened against sparse-matrix screens composed of a range of known crystallization conditions at 20 and 4°C (Jancarik & Kim, 1991 ▶). Diffraction-quality crystals were grown in a condition consisting of 0.04 *M* potassium phosphate, 16%(*w*/*v*) PEG 8000, 20%(*v*/*v*) glycerol. Crystals of SeMet PatG-DUF_sp._ grew using the same condition as the native protein. A single SeMet PatG-DUF_sp._ crystal was cryoprotected in mother liquor and flash-cooled in liquid nitrogen. A single-wavelength anomalous dispersion (SAD) data set was collected at the Se *K* absorption edge at 100 K on beamline I02 at Diamond Light Source. The structure was solved using *AutoSol* and the chains were built into electron density using *AutoBuild* from the *PHENIX* crystallography suite (Adams *et al.*, 2010 ▶). Examination of the identified anomalous scattering atoms revealed the Se atoms of the four methionine residues as expected, but also an additional atom at the interface between one monomer and its symmetry mate. The atom was coordinated by two histidine residues, an aspartic acid and a glutamic acid. Based on the anomalous scattering and coordination, we assign this atom as a Zn^2+^ ion. A map using the phases calculated from the anomalous scattering atoms and a map after density modification of these phases are shown in Supplementary Fig. S2. The structure was manually rebuilt in *Coot* (Emsley & Cowtan, 2004 ▶) and refined using *REFMAC*5 (Murshudov *et al.*, 2011 ▶). TLS restraints were generated for refinement using the *TLSMD* server (Painter & Merritt, 2006 ▶) and the structure was validated using *MolProbity* (Chen *et al.*, 2010 ▶). The Zn^2+^ ion refined with a temperature factor similar to those of the ligating atoms and no additional *F*_o_ − *F*_c_ density was observed at the site, supporting our identification of the atom as a Zn^2+^ ion. Full structural statistics are given in Table 1 ▶.

All sequence alignments were created using *Clustal Omega* (Sievers *et al.*, 2014 ▶) and presented using *ALINE* (Bond & Schüttelkopf, 2009 ▶). All structural images were generated using *PyMOL* (DeLano, 2002 ▶).

### 2.6. Expression and purification of ^15^N PatE and PatD   

For biochemical characterization an engineered PatE variant was used denoted PatE′. PatE′ harbours a single core peptide with the sequence ITACITFC (corresponding to the natural product patellamide D) and a C-terminal His_6_ tag. PatE′ and ^15^N-labelled PatE′ were produced as described previously (Koehnke *et al.*, 2013 ▶). The enzyme PatD was purified in the same way as previously described for its homologue TruD (Koehnke *et al.*, 2013 ▶).

### 2.7. Heterocyclization of PatE with PatD   

^15^N PatE′ (100 µ*M*) was incubated with PatD (5 µ*M*) in PatE′ gel-filtration buffer (10 m*M* HEPES pH 7.4, 150 m*M* NaCl, 1 m*M* TCEP) and the product was purified using size-exclusion chromatography as described previously (Koehnke *et al.*, 2013 ▶). The reaction was monitored using MALDI-TOF MS as described previously (Koehnke *et al.*, 2013 ▶).

### 2.8. ITC and data analysis   

ITC experiments were performed using a VP-ITC instrument (MicroCal) in PatE′ gel-filtration buffer at 20°C. Both PatE′ and PatG-DUF*_di_* were dialyzed into freshly prepared PatE′ gel-filtration buffer for 2 d (changing to fresh buffer after the first day) at 4°C to ensure that the buffers for the experiment were matched. A cell solution of 40 µ*M* PatG-DUF*_di_* and a syringe solution of 600 µ*M* PatE′ were prepared (using dialysis buffer to dilute the samples) and degassed at 18°C for 15 min. The PatE′ solution was titrated into the PatG-DUF*_di_* solution as follows: an initial injection of 2 µl followed by injections of 5 µl at a speed of 0.5 µl min^−1^ with a delay of 4 min between injections. The syringe was stirred at 307 rev min^−1^ for the duration of the experiment. The raw data were processed using the MicroCal *Origin* software. The baseline was adjusted and the integrations were performed manually.

### 2.9. NMR binding experiments   

NMR experiments were performed at 10°C in a Bruker DRX500 spectrometer equipped with a 5 mm TXIz probe. The instrument was run using the *TopSpin* software (Bruker). The sample for binding experiments consisted of 100 µ*M* ^15^N PatE′ in PatE′ gel-filtration buffer supplemented with 5% D_2_O. and a ^1^H–^15^N HSQC spectrum was recorded. Aliquots of 1.6 m*M* PatG-DUF*_di_* were added to final concentrations of 50, 100 and 200 µ*M* and HSQCs were recorded after each addition. The spectra were overlaid using *TopSpin* to identify changes (if any) in the spectra subsequent to the addition of PatG-DUF*_di_*. The ^1^H–^15^N HSQC spectra were acquired with Watergate water suppression (Piotto *et al.*, 1992 ▶) at 1024 × 128 points and a digital resolution of 9.8 and 19.0 Hz for the ^1^H and ^15^N dimensions, respectively. The experiment was repeated using the modified ^15^N PatE′ containing four heterocycles under the same conditions. This resulted in a poorer signal-to-noise ratio, and therefore the experiment was repeated using 250 µ*M* modified ^15^N PatE′ and aliquots of 2 m*M* PatG-DUF*_di_* were added to final concentrations of 125, 250 and 500 µ*M*.

## 3. Results   

### 3.1. Crystal structure of PatG-DUF   

The gene encoding PatG-DUF_sp._ (amino-acid residues 914–1191) was amplified from gDNA isolated from *Prochloron* sp. Since we cannot be certain as to whether the variations between PatG-DUF_sp._ and PatG-DUF*_di_* reflect natural variation amongst *Prochloron* species or have arisen from errors in the original amplification, we used site-directed mutagenesis to generate PatG-DUF with the sequence from *P. didemni*. Both PatG-DUF_sp._ (Glu958, Arg959 and Val1037) and PatG-DUF*_di_* (Asp958, Tyr959 and Met1037) were purified as described in §[Sec sec2]2 and a near-UV CD spectrum revealed no significant difference (Supplementary Fig. S3). We concluded that either sequence was suitable for use in structural characterization. Crystallography was carried out on PatG-DUF_sp._ as it had been purified first and gave crystals. We carried out biophysical studies with PatG-DUF*_di_* as we felt that these data would be more informative with a known database match.

**Figure 3:**
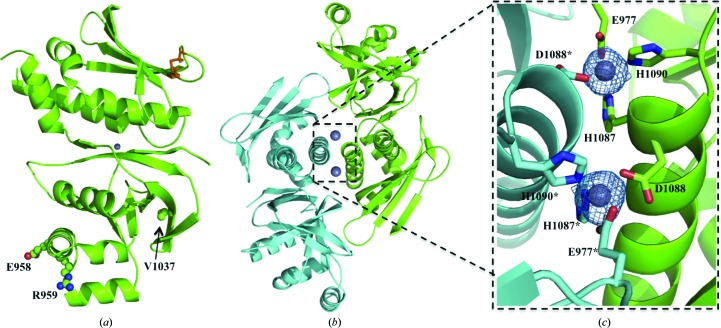
(*a*) X-ray crystal structure of PatG-DUF_sp._ represented as a cartoon. The disulfide bond between Cys1136 and Cys1142 is shown as orange sticks and the Zn^2+^ ion is shown as a grey sphere. PatG-DUF_sp._ amino acids which differ from those in PatG-DUF*_di_* are highlighted in ball-and-stick representation for clarity. (*b*) X-ray crystal structure of the PatG-DUF_sp._ dimer represented as a cartoon. (*c*) Enlargement of the Zn^2+^-coordination site. Glu977, His1087, Asp1088 and His1090 are shown as green sticks and Glu977*, His1087*, Asp1088* and His1090* are shown as cyan sticks. Difference electron density (*F*_o_ − *F*_c_) contoured at 3σ with phases calculated from a model which was refined without Zn^2+^ present is shown as a blue isomesh.

**Figure 4:**
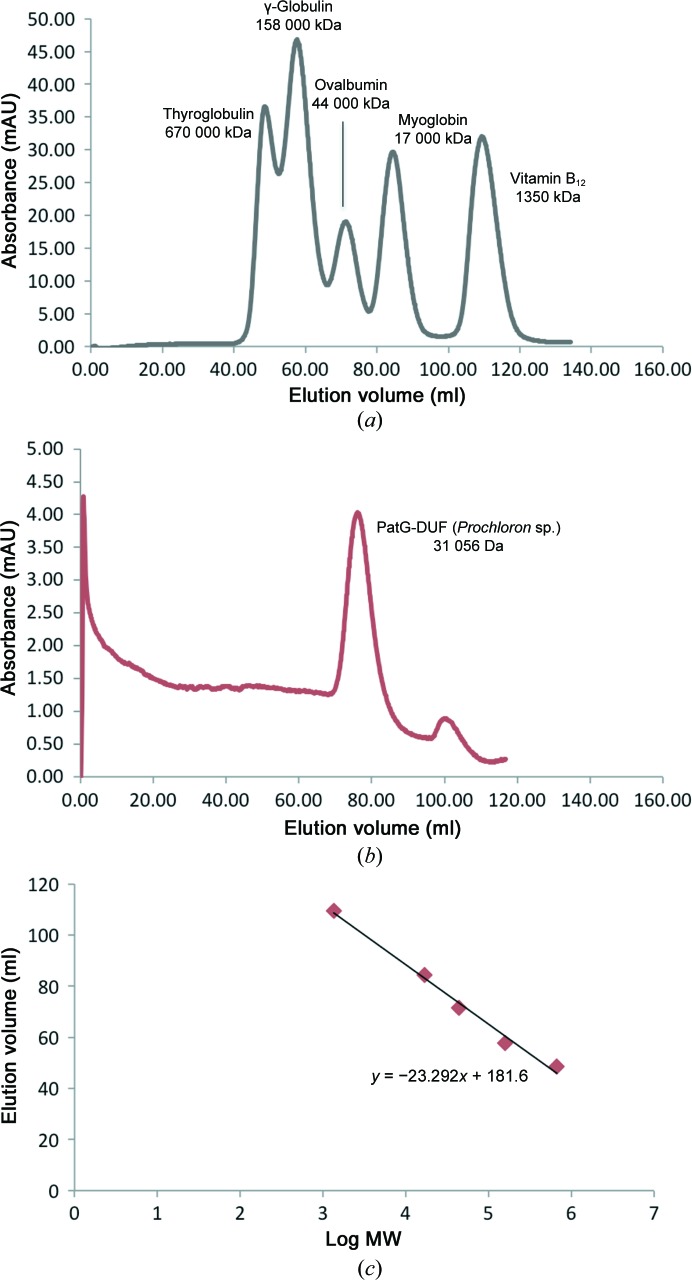
(*a*) Superdex 75 gel-filtration chromatogram of gel-filtration standards (Bio-Rad). (*b*) Superdex 75 gel-filtration chromatogram of PatG-DUF. (*c*) Standard curve (elution volume *versus* log MW). Interpolating from the standard curve, we can determine that PatG-DUF elutes from the gel-filtration column as a monomer.

The absence of PatG-DUF homologues in the PDB required the generation of a SeMet PatG-DUF_*sp.*_ derivative, and the crystal structure was solved using single-wavelength anomalous dispersion. The SeMet PatG-DUF_sp._ crystal belonged to space group *P*2_1_2_1_2 and contained one monomer in the asymmetric unit. The structure has a fold comprising seven α-helices and 13 β-strands. Analysis with secondary-structure matching at the European Bioinformatics Institute (http://www.ebi.ac.uk/msd-srv/ssm/) and *TopSearch* (https://topsearch.services.came.sbg.ac.at) both suggest that the fold is novel, with the closest matches being a series of unrelated enterotoxins giving r.m.s.d. values of between 3 and 4 Å over ∼80 residues (Krissinel & Henrick, 2004 ▶; Wiederstein *et al.*, 2014 ▶). The refined model contains residues 920–1064 and 1077–1184 (the residue numbers correspond to full-length PatG). The missing residues are at the N- and C-termini of the model and in a connecting loop between α-helices 5 and 6, and are presumed to be disordered. The model includes one disulfide bond between Cys1136 and Cys1142 and a single Zn^2+^ ion (Fig. 3 ▶ *a*). Glu958 and Arg959 are located on the opposite face from the Zn^2+^ ion, whilst Val1037 is located on the edge of a hydrophobic pocket remote from the zinc (Fig. 3 ▶ *a*). The changes in sequence would seem to be unlikely to alter the structure, which is consistent with the near-UV CD spectra. Since zinc was not deliberately added to any of the purification buffers or crystallization conditions, it is presumed the Zn^2+^ ion was bound during protein expression. The Zn^2+^ ion is coordinated in a tetrahedral arrangement by residues Glu977, His1087 and His1090. The fourth coordination site is occupied by residue Asp1088* from a symmetry mate (PatG-DUF_sp._*), creating a dimer with two Zn^2+^-binding sites (Figs. 3 ▶ *b* and 3 ▶ *c*). Analysis with *PISA* (http://www.ebi.ac.uk/pdbe/pisa/; Krissinel & Henrick, 2007 ▶) reveals that there are almost no contacts between the protein residues, and removing the Zn^2+^ ions *in silico* reduces the complex formation significance score to 0.056, indicating that Zn^2+^ is crucial in mediating dimer interactions. Since Zn^2+^ was not added, its presence in the crystal could suggest that the dimer is present in solution. However, PatG-DUF (both PatG-DUF_sp._ and PatG-DUF*_di_*) eluted from the gel-filtration column with a retention time consistent with a monomer (Fig. 4 ▶), preventing us from a firm assignment of dimer *versus* monomer in solution. Interestingly, the residues involved in Zn^2+^ binding are not all conserved in PatA-DUF (Fig. 1 ▶ *a*). While the H1087Y substitution might still coordinate Zn^2+^, the H1090R substitution would not. Additionally, these residues are not well conserved in the majority of the PatG-DUF homologues (with notable exceptions being TruG-DUF and ArtG-DUF) or in any of the PatA-DUF homologues (Supplementary Fig. S1). Consequently, we cannot make any claims regarding the biological relevance of a dimeric assembly for the DUF domain.

The structure of PatG-DUF_sp._ is novel and therefore provides no immediate insight into its function within patellamide biosynthesis. In an attempt to determine whether the C-terminal DUFs are directly involved in the biosynthesis, binding experiments between PatG-DUF*_di_* and the precursor peptide PatE′, and a modified precursor subsequent to heterocyclization by PatD, were performed using ITC and NMR.

### 3.2. Investigating potential binding partners for PatG-DUF*_di_*   

**Figure 5:**
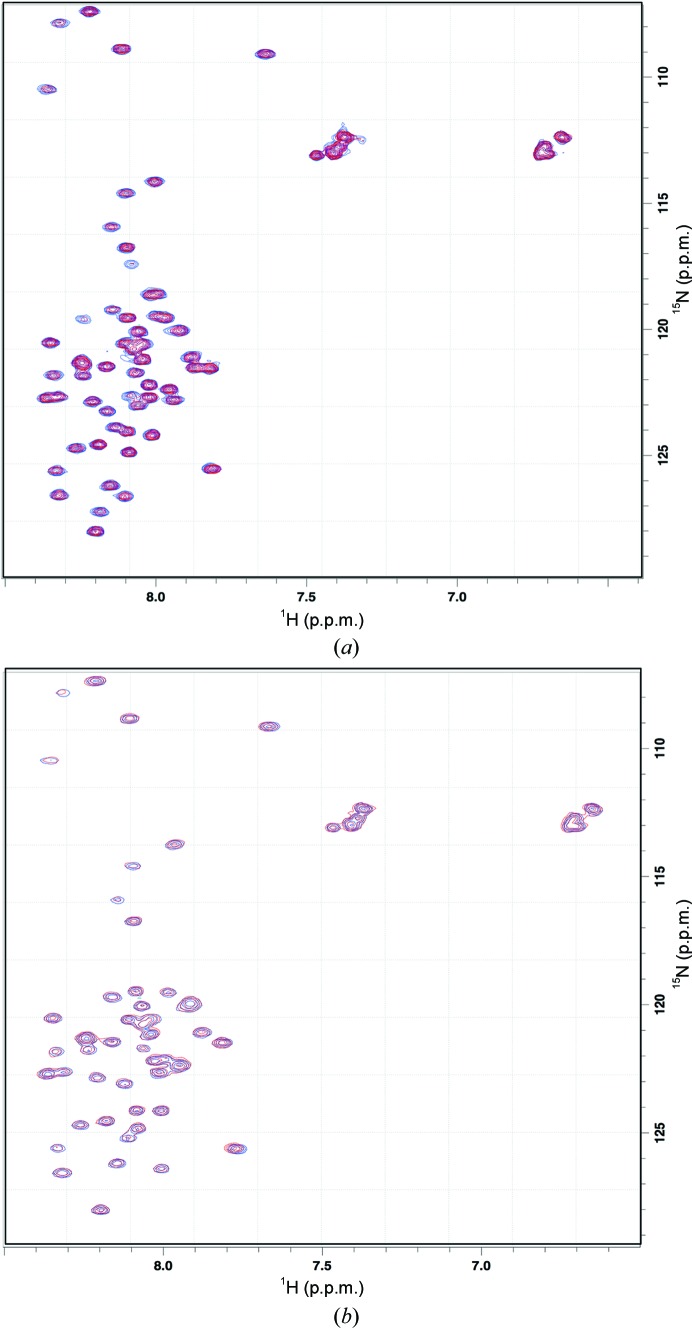
(*a*) Overlaid ^1^H–^15^N HSQC NMR spectra of PatE′ before (blue) and after (red) the addition of 2.0 equivalents of PatG-DUF*_di_*. (*b*) Overlaid ^1^H–^15^N HSQC NMR spectra of heterocyclized PatE′ before (blue) and after (red) the addition of 2.0 equivalents of PatG-DUF*_di_*.

Titrating unmodified PatE′ into a solution of PatG-DUF*_di_* reproducibly results in a binding curve (Supplementary Fig. S4). However, analysis of the data yields unlikely stoichiometries of around ten molecules of PatG-DUF*_di_* binding to one molecule of PatE′. Close inspection of the cell revealed heavy precipitation at the end of the experiment, suggesting that we were measuring protein aggregation. Using ^15^N-labelled PatE′, a ^1^H–^15^N HSQC spectrum was recorded in the absence and presence of 0.5, 1.0 and 2.0 molar equivalents of PatG-DUF*_di_* (Supplementary Fig. S5*a*). The spectra of pure ^15^N PatE′ in the absence (blue) and presence (red) of 2.0 equivalents of PatG-DUF*_di_* have been overlaid (Fig. 5 ▶ *a*), and clearly show an identical chemical shift pattern, showing that PatG-DUF*_di_* does not bind the unmodified precursor peptide. The NMR experiments were repeated using ^15^N PatE′ that had been processed with PatD, introducing four heterocycles (two thiazolines and two oxazolines) within the context of the core peptide. As before, the addition of up to 2.0 equivalents of PatG-DUF*_di_* to the modified PatE′ resulted in an identical NMR spectrum (Fig. 5 ▶ *b* and Supplementary Fig. S5*b*), indicating that the heterocycle-containing peptide does not bind PatG-DUF*_di_* either. The known disorder of the leader peptide of PatE (Koehnke *et al.*, 2013 ▶) would suggest that blocking of PatG-DUF*_di_* binding by the leader peptide is unlikely. At present, we are unable to produce labelled cleaved precursor or macrocycle in sufficient quantities and purity for NMR experiments.

## 4. Conclusions   

The patellamide proteins PatA and PatG contain C-terminal domains of unknown function which are 56% identical to each other and are conserved in related cyanobactin biosynthetic pathways. X-ray crystallography revealed the structure of PatG-DUF_sp._ to be a novel fold and, although a dimer is observed in the crystal, the residues involved in Zn^2+^ coordination (which is required for dimerization) are not well conserved in PatA-DUF or in homologues from other pathways. Consequently, it is difficult to speculate as to the functional importance of the dimer. We have been unable to detect any binding to linear substrates (simple peptides or peptides with heterocycles), indicating that these are unlikely to be substrates or ligands of the DUFs. The pitfall in using one technique to assess binding was shown by the reproducible but functionally meaningless ITC binding curves that we observed. Further study is required with macrocycles before we can rule out any binding of the DUF domain to patellamide substrates. It remains possible that the dimerization of the domain indicates an interaction between the DUFs, potentially leading to homodimers of PatA/PatG or even heterodimers of PatA and PatG, which might be important for the activity of these enzymes *in vivo*. Further study is required to investigate such hypotheses. If the DUF domain has no enzymatic role, coupled with the observation that the protease domains of PatA and PatG are active in isolation, it is interesting as to why it is conserved throughout cyanobactin biosynthetic pathways. However, it also suggests that it could be excluded from *in vitro* enzyme-based synthesis systems. This simplifies the design and use of a biotechnological ‘toolkit’ for bioactive cyclic and linear peptides.

## Supplementary Material

PDB reference: PatG-DUF, 4uvq

Supplementary figures.. DOI: 10.1107/S2053230X1402425X/sx5117sup1.pdf

## Figures and Tables

**Table 1:** Data-collection and refinement statistics for SeMet PatG-DUF_sp._ Anomalous data were collected using a single crystal on beamline I02 at the Diamond Light Source. Statistics are average values; values for the highest resolution shell are given in parentheses.

Data collection	
Wavelength ()	0.9797
Space group	*P*2_1_2_1_2
Unit-cell parameters (, )	*a* = 64.1, *b* = 96.2, *c* = 40.2, = = = 90.0
Resolution ()	53.171.72 (1.771.72)
*I*/(*I*)	12.1 (3.4)
*R*_merge_ (%)	14.5 (89.6)
Completeness (%)	99.9 (100)
Multiplicity	14.0 (11.3)
Anomalous completeness (%)	99.9 (99.9)
Anomalous multiplicity	7.3 (5.7)
Initial map correlation	0.4
Refinement	
*R* factor (%)	17.5 (23.9)
*R*_free_ (%)	20.0 (29.5)
R.m.s.d., bond lengths ()	0.005
R.m.s.d., bond angles ()	0.932
No. of non-H atoms	
Protein atoms	2109
Solvent atoms	269
Heterogen atoms	1
*B* factors (^2^)	
All	26.0
Protein	25.1
Ligand (Zn^2+^)	12.9
Water	32.8

## Footnotes

1 Supporting information has been deposited in the IUCr electronic archive (Reference: SX5117).
