# Supplementary material for: The structure of the cyanobactin domain of unknown function from PatG in the patellamide gene cluster
Source: Acta Crystallogr F Struct Biol Commun. 2014 Nov 14;70(Pt 12):1597–603. doi: 10.1107/S2053230X1402425X (PMC4259220; doi:10.1107/S2053230X1402425X)
Supplement: Supplementary file 1 [file f-70-01597-sup1.pdf]

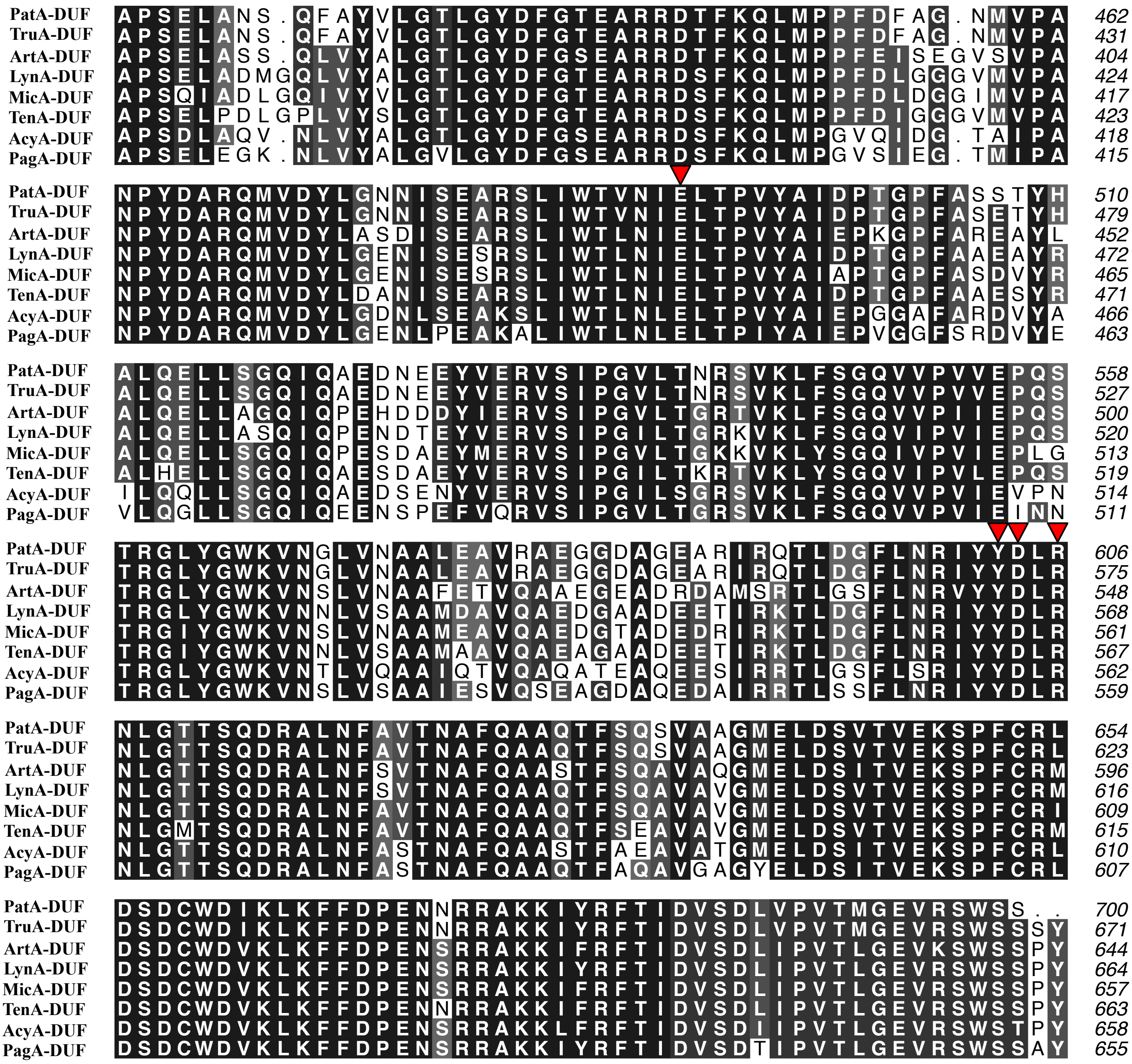

Fig. S1A Sequence alignment between PatA-DUF and homologues from related cyanobactin biosynthetic pathways. Residues involved in Zn^{2+} coordination (as identified in the PatG-DUF$_{sp.}$ structure) are highlighted with red triangles.

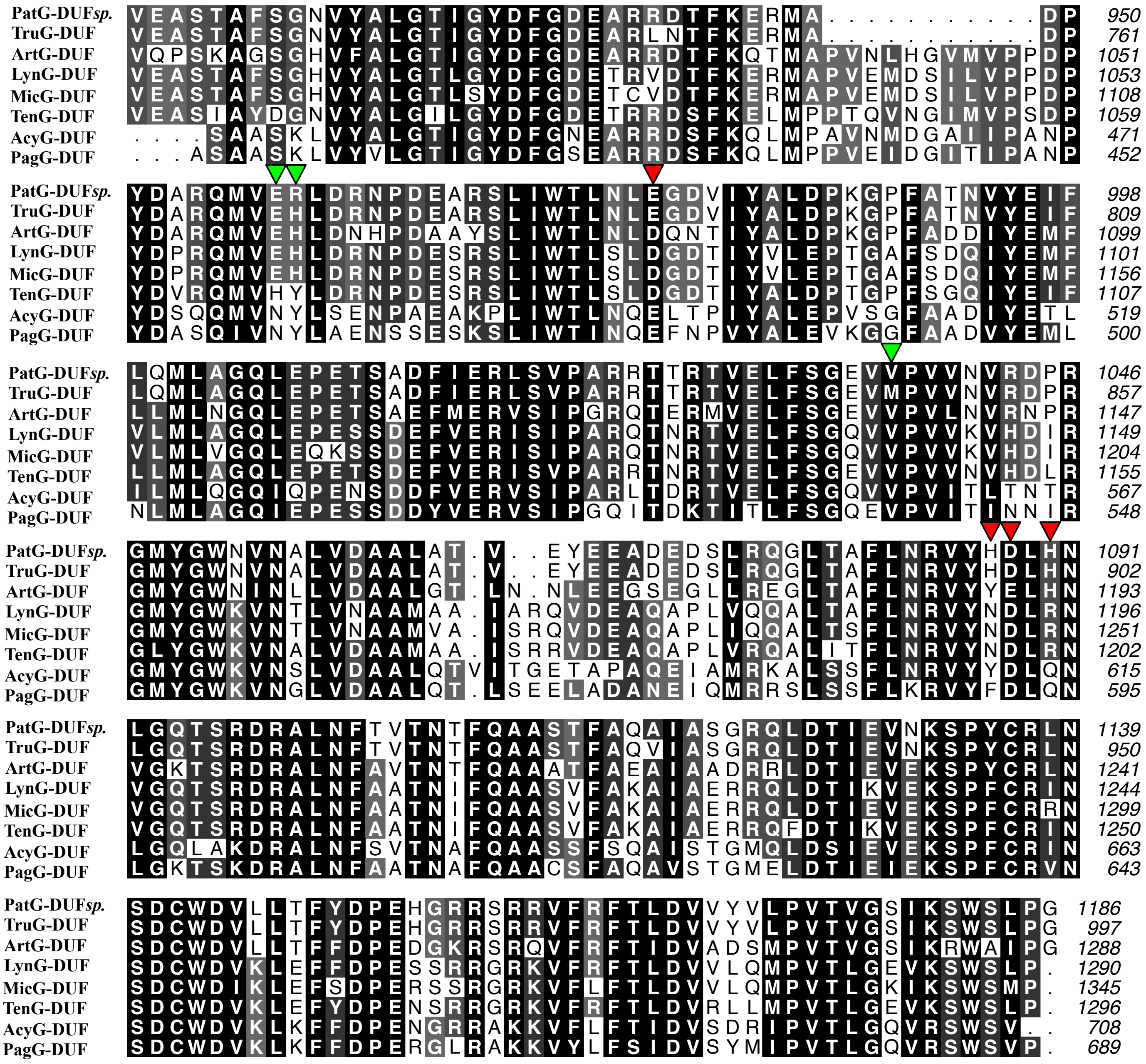

Fig. S1B Sequence alignment between PatAG-DUF$_{sp.}$ and homologues from related cyanobactin biosynthetic pathways. Residues involved in Zn^{2+} coordination (as identified in the PatG-DUF$_{sp.}$ structure) are highlighted with red triangles.

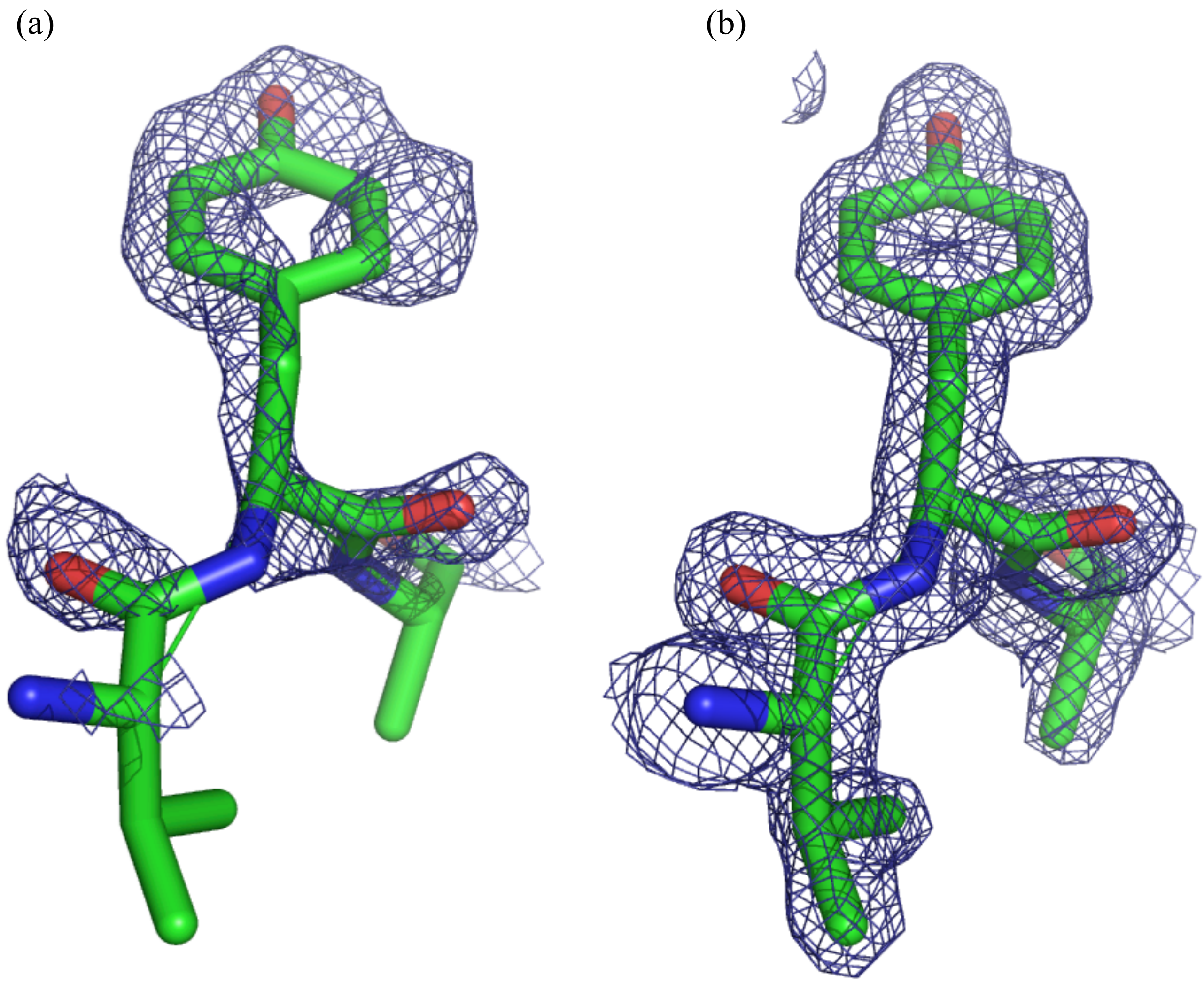

Fig. S2 Final refined of PatG-DUF$_{sp.}$ Y925 model with (a) Electron density map contoured at 1.2 σ from PHENIX with experimental phases calculated from anomalous scattering atoms. (b) Electron density map calculated with phases after density PHENIX modification of experimental phases.

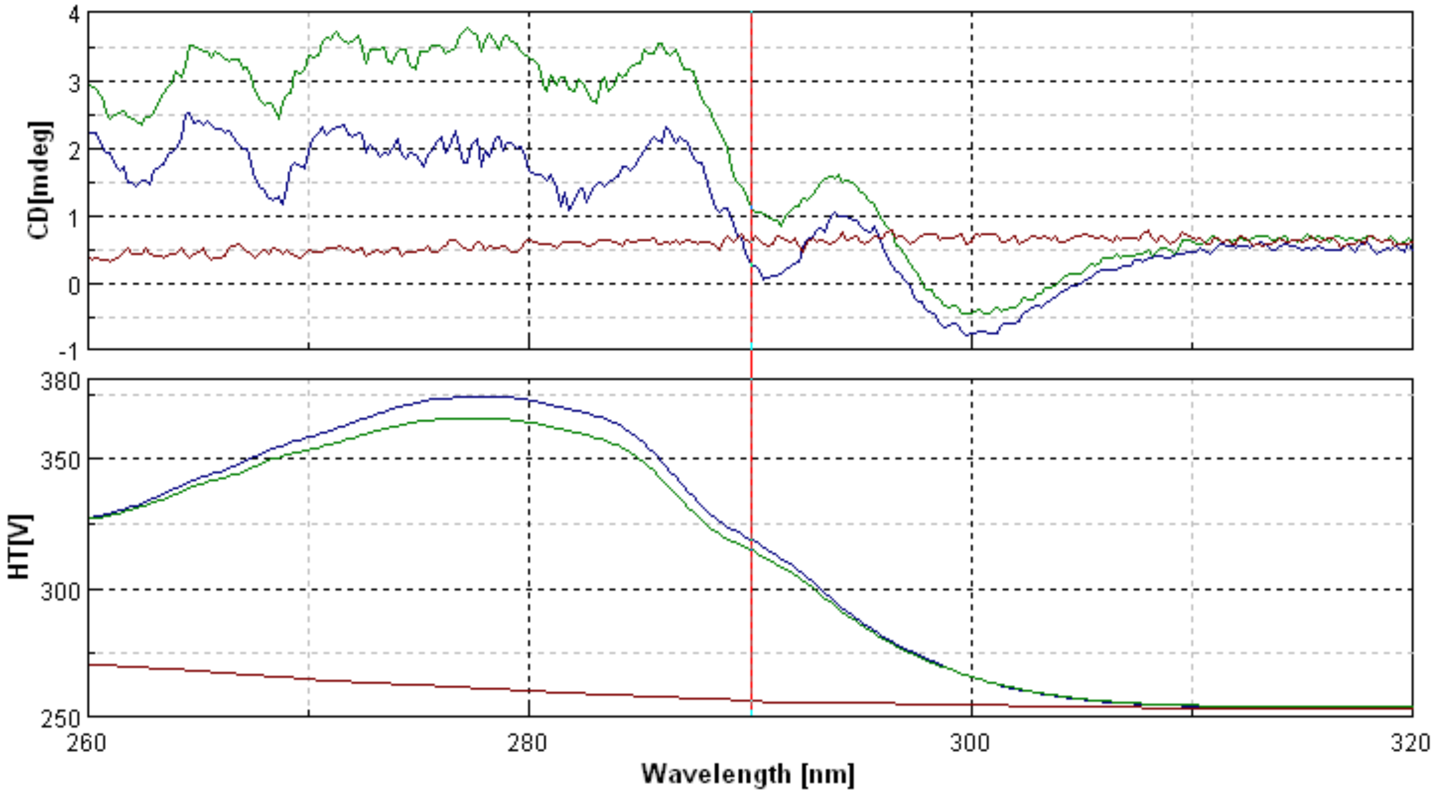

Fig. S3 Near UV CD-spectra of PatG-DUF$_{sp.}$ (green) and PatG-DUF$_{di.}$ (blue). A blank containing only buffer is shown in red. The CD-spectra overlay well suggestion the sequence variations (three point mutations) do not alter the tertiary structure of the domain.

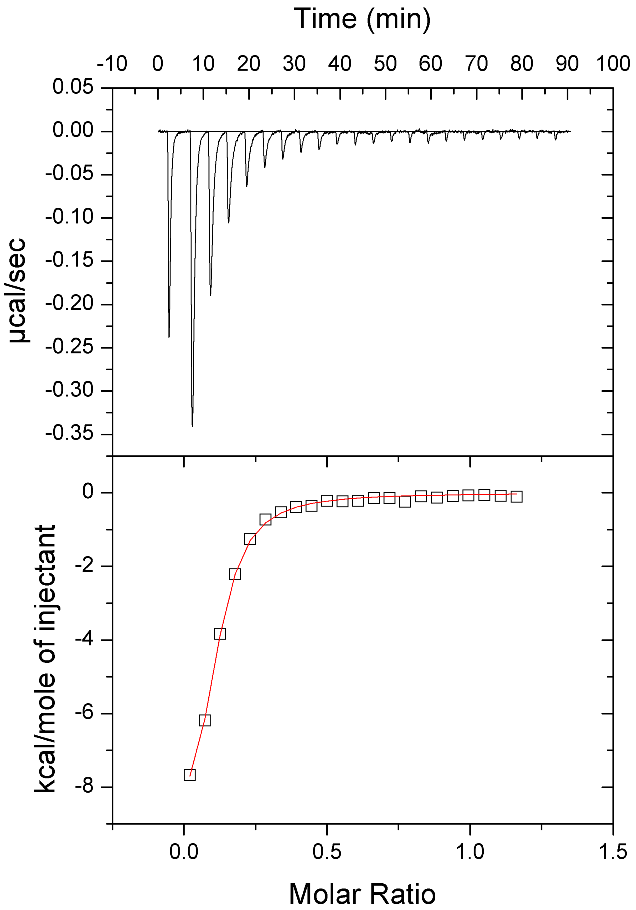

Fig. S4 ITC data of PatE' titrated into PatG-DUF$_{di.}$ solution. The top panel shows raw data representing the heat evolved in response to injections, the bottom panel shows the integrated heats of injections (□) and the best fit (-) to the one-site model (origin).

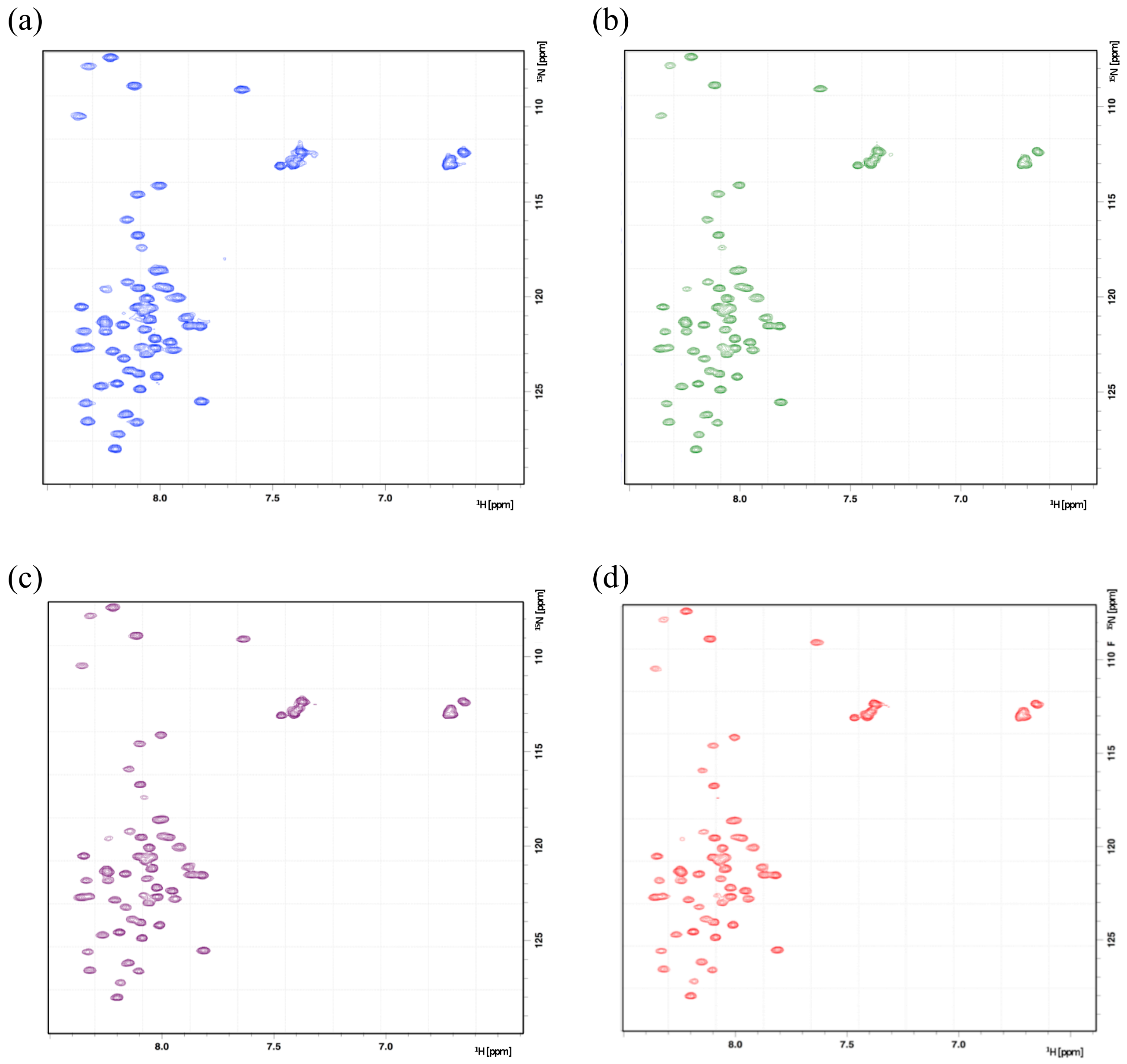

Fig. S5A ^{1}H-^{15}N-HSQC of PatE' (blue) with 0.5 (green), 1.0 (purple) and 2.0 (red) equivalence of PatG-DUF$_{di.}$

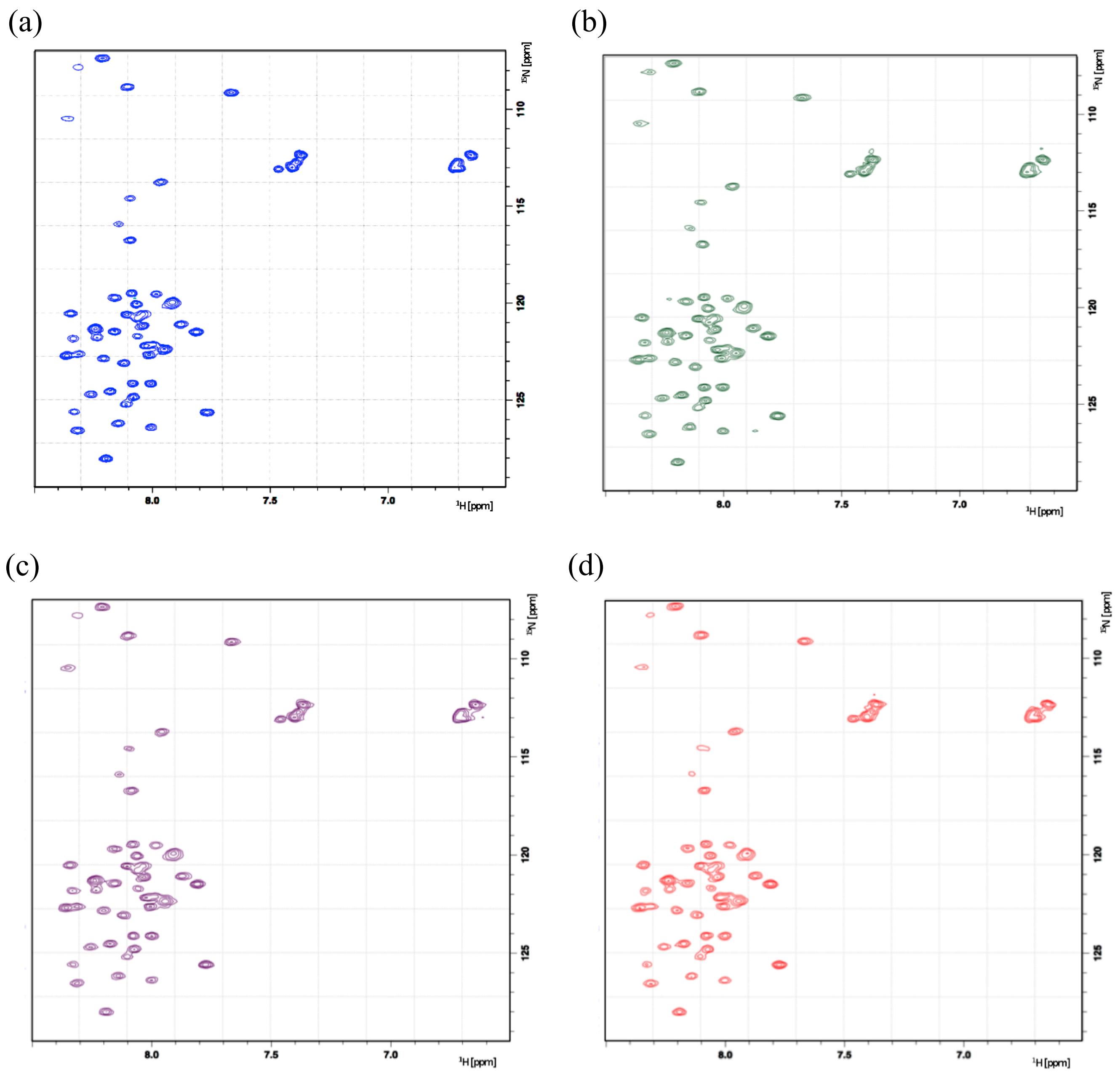

Fig. S5B ^{1}H-^{15}N-HSQC of heterocyclized-PatE' (blue) with 0.5 (green), 1.0 (purple) and 2.0 (red) equivalence of PatG-DUF$_{di.}$
